# Editorial: The role of metabolic syndrome and disorders in cardiovascular disease

**DOI:** 10.3389/fendo.2023.1327394

**Published:** 2023-10-31

**Authors:** Paola Di Pietro, Carmine Izzo, Albino Carrizzo

**Affiliations:** ^1^ Department of Medicine, Surgery and Dentistry “Scuola Medica Salernitana”, University of Salerno, Baronissi (SA), Italy; ^2^ Vascular Physiopathology Unit, IRCCS Neuromed, Pozzilli (IS), Italy

**Keywords:** metabolic syndrome, risk factors, cardiovascular disease, biomarkers, cardiovascular complications

Metabolic syndrome (MetS) is a cluster of interrelated risk factors that includes abdominal obesity, insulin resistance, hypertriglyceridemia, and arterial hypertension and is strongly associated with an increased risk for developing atherosclerotic cardiovascular disease, diabetes mellitus, and vascular and neurological complications.

The present Research Topic, entitled “*The Role of Metabolic Syndrome and Disorders in Cardiovascular Disease”*, aims at highlighting the risk factors predisposing to MetS and its related cardiovascular complications.

The prevalence of metabolic diseases has drastically risen worldwide over the last decades (1, 2). Using National Health and Nutrition Examination Survey (NHANES) data from 1999 to 2014, Li et al. evaluate trends in MetS prevalence among US adults, showing a significant increase from 27.6% to 32.3% and, a strong gender differences, with a higher prevalence but a lower risk in women compared with men.

In accordance with other recent papers (3), the study from Cai et al. confirms the impact of obesity on the risk of hypertension in a community-based cohort study. According to the inclusion criteria, the authors analyzed a total of 2,618 subjects free from hypertension at baseline examination. After nearly 7 years of follow-up, the authors show that keeping obese status all the time increased the risk of hypertension by 30%. On the other side, losing weight after being obese was associated with a higher risk of hypertension than changing weight from normal to obesity, highlighting the importance of weight management as a preventive measure against hypertension. Interestingly, there was an inverse correlation between female subjects and increased incidence of hypertension, whereas a positive correlation was found in individuals aged more than 60 years.

In the same order of ideas, Tan et al. conduct a *post hoc* analyses of data from the TOPCAT trial (4) to explore the effect of body weight fluctuations on the prognosis in patients with heart failure and preserved ejection fraction (HFpEF). Patients were grouped in quartiles according to the variation of both BMI and waist circumference (WC), and primary endpoint, CVD death, and hospitalization for HF were evaluated as outcomes. Over a mean follow-up of 3.3 years, variability of both BMI and WC was associated with poor prognosis of patient with HFpEF, and the risk of clinical adverse events increased with increasing variability.

In the effort to improve the management of patients with obesity and cardiovascular diseases (CVD), the meta-analysis study by Zhang et al. reports excessive BMI as an independent risk factor for preoperative oxygenation impairment in patients with acute aortic syndrome (AAS). These findings were further supported by a retrospective study including a total of 230 individuals, demonstrating a significant higher risk of preoperative oxygenation impairment in those with BMI of 25 kg/m^2^ or greater. Besides, these authors observed that the risk of AAS with preoperative oxygenation impairment increased dramatically with the increased BMI, thus suggesting the potential value of BMI as an indicator for risk stratification in AAS patients.

Another important contribution to the evaluation on the influence of obesity on cardiovascular complications has been provided by two papers collected in the current Research Topic.

Although obesity has long been defined as elevated BMI, growing evidence demonstrates that subjects with similar BMIs may have different CVD risk profiles. Thus, susceptibility to cardiovascular complications is dependent upon individual differences in regional body fat distribution rather than the amount of adipose tissue (5).

In line with this notion, Du et al. evaluated the predictive value of three novel obesity indices for the detection of cardiovascular subclinical organ damage (SOD) in the general Chinese population. To clarify this issue, they examined a total of 1,773 healthy Chinese subjects and analyzed lipid accumulation product (LAP), visceral adiposity index (VAI), and triglyceride-glucose (TyG), which are the product of waist circumference, BMI and blood lipid profile. The authors found a significant positive association of these indices with arterial stiffness and albuminuria, with the TyG representing the most discriminating index in identifying arterial stiffness and albuminuria, as compared with the other two indices. These data open a new avenue for the development of novel preventive approaches against cardiovascular SOD progression and adverse cardiovascular outcomes.

People with metabolic syndrome are at higher risk to develop stroke, with the highest percentage of stroke being in people over 56 years old (6). Using data from the NHANES database, the study from Chen et al. explores the association between VAI and stroke prevalence among US adults, showing that higher VAI was associated with higher stroke prevalence and report for the first time a negative correlation with age at stroke.

Since metabolic syndrome has emerged as a nonclassical complication of primary hyperparathyroidism (PHPT), with the aim at identifying unknown mechanisms underlying the link between endocrine-metabolic disorders and related-cardiac abnormalities, in their study Chen et al. examine the relationship between primary hyperparathyroidism and cardiac dysfunction. After adjustment for age, gender, BMI, duration of PHPT, hypertension, and diabetes, these authors showed that calcium and parathyroid hormone levels were positively correlated with left ventricular mass index, whereas an inverse correlation was found with diastolic dysfunction, as indicated by the decreased early diastolic mitral inflow velocity index.

Another paper by Wang et al. reports the influence of thyroid hormone levels on cardiac function. Interestingly, in euthyroid patients with valvular heart disease, thyroxine (T4) and triiodothyronine (T3) levels were significantly decreased in proportion with increasing NYHA grades. Further studies are warranted to explore the potential of hormone replacement therapy to improve clinical outcomes in patients with valvular disease.

An interesting study from Pillai et al. investigates the impact of primary hyperaldosteronism (PA) in patients with diabetes and hypertension. Using data from the National Impatient Sample, these authors demonstrated a strong association between the presence of PA in hypertensive and diabetic individuals and increased mortality and morbidity.

Yao et al. examine characteristics and risk of CVD among 1,765 Chinese individuals with diabetes mellitus, reporting that age at diagnosis, diabetes duration, hypertension and hyperlipidemia were independent risks of CVD. Further, a longer duration of diabetes (>15 years) increased the 10-year ASCVD risk prediction.

Liu et al. demonstrate the ability of estimated glucose disposal rate (eGDR) to independently predict in-stent restenosis (ISR) after percutaneous coronary intervention (PCI) in patients with non-ST-segment elevation acute coronary syndrome (NSTE-ACS). Furthermore, eGDR improves the predictive ability of conventional cardiovascular risk factors to ISR, especially in patients without type 2 diabetes mellitus (T2DM).

Numerous biomarkers have been proposed to improve understanding of biological processes involved in MetS pathophysiology. In this context, Zalewska et al. demonstrate that patients with MetS had lower levels of catestatin (Cts). Further, Cts correlated positively with high density lipoprotein cholesterol and negatively with BMI, and 10-year atherosclerotic cardiovascular disease risk (ASCVD).

MetS has also been shown to be independently associated with an increased risk of new-onset atrial fibrillation (AF) (7). Given the evidence of a positive association of both dysglycaemia and hyperuricemia with the increased risk of atrial fibrillation (AF), Zhong et al. explore the relationship between serum uric acid (SUA) and AF in different fasting glucose (FBG) patterns. SUA and AF were independently correlated after adjusting for different FBG patterns. In addition, in patients with AF, SUA levels correlated with several metabolic factors in different FBG patterns. Although the retrospective nature of the study, this is the only manuscript yet to exist evaluating the association between SUA, dysglycaemia and AF, and offer new perspectives to better understanding the potential contribution of SUA in the pathogenesis of AF and its related predisposing factors.

Since SUA levels could be affected by renal function, a paper by Zhang et al. reports the usefulness of a renal function-normalized uric acid/creatinine ratio (UCR) as a valuable biomarker to predict the recurrence of AF after catheter ablation. After a mean follow-up of nearly 2 years, regression analysis revealed that UCR was an independent predictor of AF recurrence, and that increased preoperative UCR was associated with AF recurrence in patients with paroxysmal AF and in male patients, but not in patients with persistent AF as well as in female. Although the concept has been raised in previous reports, this study suggests a higher risk of AF recurrence in female subjects than in male. However, the sample size was relatively small and the study was performed with data from a single center, which warrants further prospective and larger studies.

Preclinical studies have reported fibroblast growth factor 21 (FGF21) as a metabolic regulator with potent beneficial effects on obesity and diabetes (8). In a meta-analysis study, Yan et al. explore the potential role of FGF21 on predicting long-term prognosis MACE among CVD patients stratified by coronary artery disease (CAD) and HF. While no association was found between blood FGF21 levels and the endpoint in HF patients, increased FGF21 levels were independently associated with the incidence of major adverse cardiovascular events and all-cause death among patients with CAD.

In the only preclinical study of this Research Topic, Shymotiuk et al. demonstrate that vitamin A (VitA), whose metabolism is impaired in patients and animal models with obesity and T2DM, plays a critical role in mediating steatosis and adverse organ remodeling in a diet-induced obesity murine model independently of altered mitochondrial metabolism.

In conclusion, the findings collected in this Research Topic (**Figure 1**) highlight the importance of improving the management of such a multidimensional risk factor syndrome. Apart from non-modifiable risk factors, interventions focused on lifestyle habits could be emphasized in patients with MetS. Treating each of the risk factors contributing to metabolic syndrome and exploring novel biomarkers could inform the development of additional preventive strategies for the diagnosis of MetS and its associated complications at earlier stages, ultimately leading to improved long-term survival outcomes.

**Figure 1:**
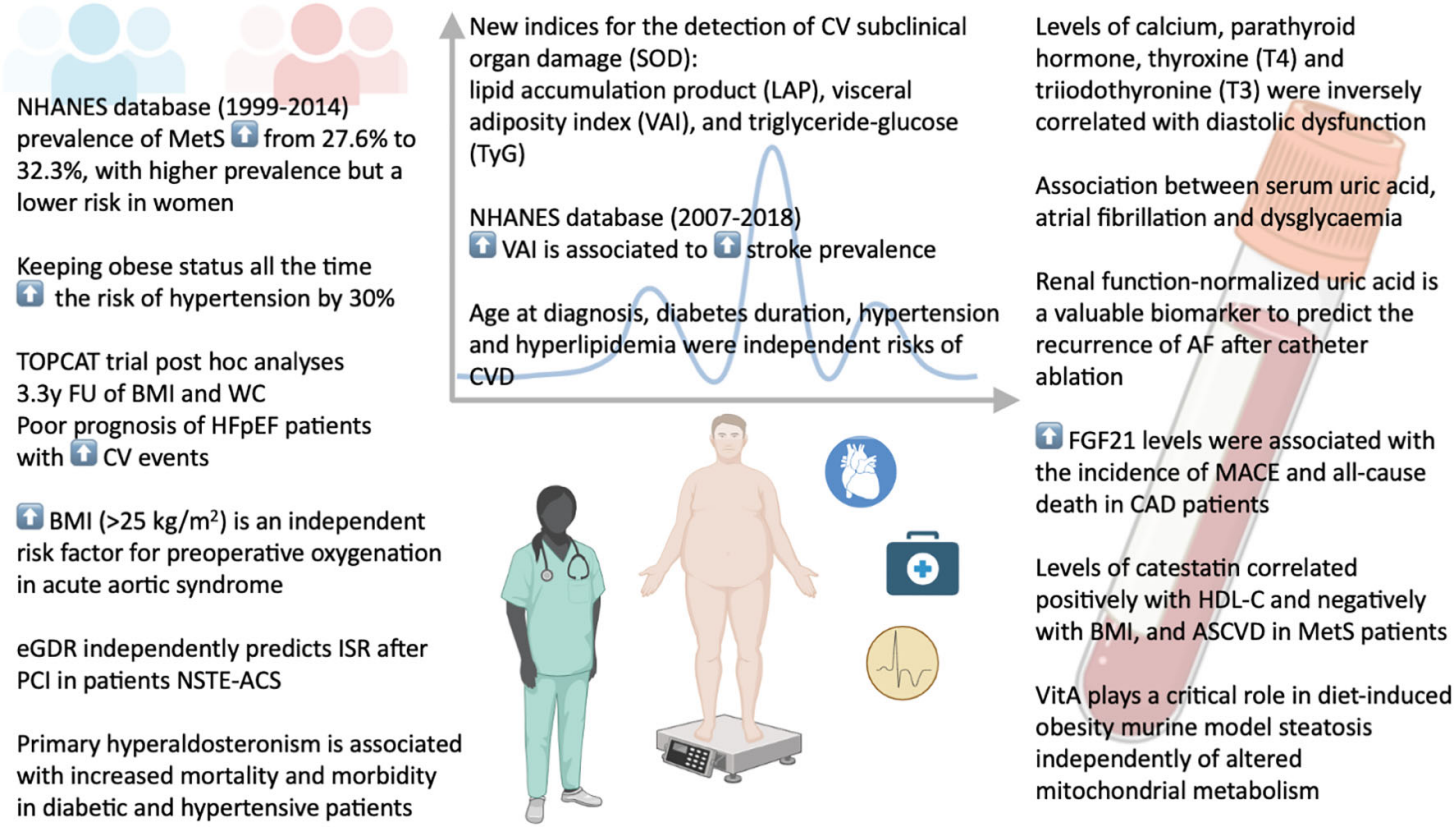
Risk factors, cardiovascular complications, and potential biomarkers in metabolic syndrome.

## Author contributions

PP: Conceptualization, Writing – original draft, Writing – review & editing. CI: Data curation, Writing – original draft. AC: Conceptualization, Writing – review & editing, Supervision.
